# Combination of Exercise and Vegetarian Diet: Relationship with High Density-Lipoprotein Cholesterol in Taiwanese Adults Based on *MTHFR* rs1801133 Polymorphism

**DOI:** 10.3390/nu12061564

**Published:** 2020-05-27

**Authors:** Shu-Lin Chang, Oswald Ndi Nfor, Chien-Chang Ho, Kuan-Jung Lee, Wen-Yu Lu, Chia-Chi Lung, Disline Manli Tantoh, Shu-Yi Hsu, Ming-Chih Chou, Yung-Po Liaw

**Affiliations:** 1 Institute of Medicine, Chung Shan Medical University, Taichung 40201, Taiwan; sherry.chang@kimo.com; 2 Department of Public Health and Institute of Public Health, Chung Shan Medical University, Taichung 40201, Taiwan; nforoswald2@yahoo.com (O.N.N.); jasminemachi@gmail.com (K.-J.L.); yvonne841026@gmail.com (W.-Y.L.); dinoljc@csmu.edu.tw (C.-C.L.); tantohdisline@yahoo.com (D.M.T.); sui0209@gmail.com (S.-Y.H.); 3 Department of Physical Education, Fu-Jen Catholic University, New Taipei City 24205, Taiwan; ccho1980@gmail.com; 4 Research and Development Center for Physical Education, Health, and Information Technology, Fu Jen Catholic University, New Taipei 24205, Taiwan; 5 Department of Medical Imaging, Chung Shan Medical University Hospital, Taichung City 40201, Taiwan

**Keywords:** lipids, diet, exercise, polymorphism, biobank

## Abstract

We examined the association between high-density lipoprotein cholesterol (HDL-C), and exercise and vegetarian diets, in Taiwanese adults, based on the *Methylenetetrahydrofolate reductase* (*MTHFR)* rs1801133 polymorphism. Using regression models, we analyzed historical data collected from 9255 Taiwan Biobank (TWB) participants from 2008 through 2015. Exposure to exercise was associated with higher HDL-C (*β* = 1.0508 and 1.4011 for GG and GA + AA individuals, respectively), whereas a vegetarian diet was associated with lower HDL-C (*β* = −6.2793 and −4.6359 for those with GG and GA + AA genotype, respectively). We found an interaction between exercise and diet among GG individuals (*p* = 0.0101). Compared with no exercise/no vegetarian diet, vegetarian diet/no exercise was associated with a 5.1514 mg/dl reduction in HDL-C among those with GG genotype (*β* = −5.1514, *p* < 0.0001) and a 4.8426 mg/dl reduction (*β* = −4.8426, *p* < 0.0001) among those with GA + AA genotype. Vegetarian diets in combination with exercise predicted a 6.5552 mg/dl reduction in HDL-C among GG individuals (*β* = −6.5552) and a 2.8668 mg/dl reduction among GA + AA individuals (*p* < 0.05). These findings demonstrated that vegetarian diet alone was associated with lower HDL-C, no matter the rs1801133 genotype. However, the inclusion of regular exercise predicted much lower levels among GG individuals, whereas levels among GA + AA individuals were relatively higher.

## 1. Introduction

High-density lipoprotein cholesterol is a well-known lipid fraction [1] that has been associated with the risk of heart diseases [2]. Lower levels of HDL-C (i.e., <40 mg/dl and <50 mg/dl for men and women, respectively) have been associated with heightened risk of cardiovascular disease, even though the epidemiological relationship between them remains complex [3]. Lower concentrations of HDL-C have also been associated with systemic inflammation and elevated risk of sepsis [4,5]. 

Vegetarian diets are important in the prevention and treatment of diseases [6]. However, previous works investigating lipid profiles have reported negative associations between HDL-C and vegetarian diets [7,8,9]. In a separate study, consumption of vegetarian diets was associated with decreased levels of HDL-C, low-density lipoprotein cholesterol (LDL-C), and total cholesterol (TC), but not with triglycerides (TG) [10]. In addition, vegetarian diets have also shown strong associations with total plasma homocysteine (Hcy) levels [11]. 

Exercise is among the lifestyle variables previously recommended for boosting lipid profiles [12]. More recent evidence [13] suggested dose–response relationships between HDL-C and exercise intensity. Habitual or a single bout of exercise can positively affect cholesterol metabolism [14]. According to MayoClinic, benefits can be seen with as little as 60 min of moderate aerobic exercise a week [15]. It has been hypothesized that exercise can increase the expression of liver ATP-binding cassette transporters A-1, which play an essential role in the reverse cholesterol transport system [16].

Exercise is believed to provide more benefits for HDL-C than those obtained by eating just vegetarian diets [17]. It is widely believed that exposure to exercise is a great way for vegetarians to boost their HDL cholesterol [15]. As far as we know, investigations have not been carried out to determine whether coupling a vegetarian diet with exercise would predict a reduction or an increase in HDL-C. Such investigations would help to enhance the knowledge of HDL-C response in terms of lifestyle variables. 

Besides the lifestyle determinants, genetic factors contribute to variations in HDL-C concentrations. The *methylenetetrahydrofolate reductase* (*MTHFR*) gene, an important enzyme for folate metabolism, has been demonstrated to be associated with abnormal lipid levels and coronary artery diseases [18,19,20]. Rs1801133 (also known as C677T) is one of its most common and best-studied polymorphisms, which showed stronger associations with cardiovascular disease risk, especially among Asians [18]. This variant reduces enzyme activity and raises the concentration of total Hcy [21], which is a potential risk factor for dyslipidemia and cardiovascular disease [22]. The significance of this polymorphic variant has already been previously described [23]. Interactions previously reported between C677T and serum lipid levels have been partly linked to lifestyle and environmental factors [24]. Of these factors, exposure to regular exercise has been demonstrated to attenuate central artery stiffening caused by the C677T variant [25].

As stated earlier, exercise and vegetarian diets are some of the modifiable factors previously associated with HDL-C. However, whether the combined effect of these variables on HDL-C is modified by rs1801133 polymorphism has not yet been established. Therefore, we examined the interactive association of exercise and vegetarian diets with HDL-C among Taiwanese individuals, based on *MTHFR* rs1801133 polymorphism. 

## 2. Methods

Data were obtained from the TWB, an established infrastructure holding data of Taiwanese individuals (aged 30–70 years) recruited from assessment centers across Taiwan, from 2008 through 2015. Before data collection, all participants signed written informed consent. Currently, TWB contains biological, experimental, questionnaire, and physical assessment data, collected from over 129,054 participants from different parts of Taiwan. We obtained ethical approval from the Institutional Review Board of Chung Shan Medical University (CS2-16114).

### 2.1. Study Participants

At the beginning of the study, data were available for 9287 TWB participants with no history of cancer. However, we excluded 32 individuals with incomplete or missing information. Finally, we analyzed data for 9255 participants (4938 women and 4317 men). Sociodemographic and clinical variables of the participants included sex, age, diet type, smoking, coffee drinking, alcohol intake, total cholesterol (TC), triglycerides (TG), low-density lipoprotein (LDL-C), body mass index (BMI), waist-hip ratio (WHR), uric acid and body fat. 

Diet type, smoking, alcohol intake and coffee consumption were assessed through self-reported questionnaires held in the TWB dataset. In our analyses, participants were grouped based on rs1801133 genotypes (i.e., GG and GA + AA) and diet type. They were classified as former vegetarians if they were not currently on vegetarian diets but reported to have consistently consumed them for at least 6 months in their lifetime. Vegetarians included participants who had continuously consumed plant-based food for 6 months or more, and were currently on the diet during the recruitment period. Regular exercise was considered as engaging in at most 3 physical activities lasting 30 min each session, and at least 3 sessions a week. The exercise patterns contained in the TWB questionnaires have already been discussed in our previous publication [26]. Details of the other lifestyle variables included in our model have also been previously described [27]. 

### 2.2. Variant Selection/Genotyping

We selected *MTHFR* rs1801133, which is one of the most common and the best-studied polymorphisms previously associated with serum lipid levels [24]. We performed genotyping using the Axiom Genome-Wide Array Plate System (Affymetrix, Santa Clara, CA, USA). For quality control purposes, we included only participants whose call rates were over 90%, and excluded single nucleotide polymorphisms (SNPs) with low minor allele frequencies (i.e., <0.05). SNPs that were not in Hardy–Weinberg equilibrium (that is, *p* < 1.0 × 10^−3^) were also excluded.

### 2.3. Statistical Analysis

PLINK 1.09 beta and SAS 9.4 software (SAS Institute, Cary, NC, USA) were used to perform analyses. We separated participants into 3 diet categories that included non-vegetarians, former vegetarians and vegetarians (including lacto-ovo-vegetarian and vegan). In total, 443 participants were vegetarians. Among them, 142 were rs1801133-GG individuals without exercise, 100 were rs1801133-GG individuals with exercise, 125 were rs1801133-GA + AA individuals without exercise, and 76 were rs1801133-GA + AA individuals with exercise. We analyzed the categorical variables using the Chi-square test and the continuous variables were described as means ± standard error. Using multivariate linear regression analysis, we estimated the β coefficients and their corresponding *p*-values. Exercise and vegetarian diets each were considered to be 1 unit of change. A statistically significant beta coefficient indicated that the variable predicted an outcome. 

## 3. Results

The baseline characteristics of participants according to rs1801133 are described in Table 1. There were 5016 individuals with the rs1801133-GG genotype and 4239 with the GG + AA genotype. Overall, vegetarian diet was associated with lower HDL-C among those with rs1801133-GG (*β* = −6.2793, *p* < 0.0001) and rs1801133-GG + AA (*β* = −4.6359, *p* < 0.0001) genotypes, respectively (Table 2). Exposure to exercise predicted a higher HDL-C among those with both genotypes, with β values of 1.0508 (*p* = 0.0015) for rs1801133-GG individuals and 1.4011 (*p* = 0.0001) for rs1801133-GG + AA individuals. Compared to women, men with rs1801133-GG and rs1801133-GG + AA genotypes were associated with lower HDL-C. Their *β* values were −7.9509 (*p* < 0.0001) and −7.2069 (*p* < 0.0001), respectively. The interaction between diet type and exercise exposure was significant only among those with the GG genotype (*p* = 0.0101). Compared with the non-vegetarian diet, vegetarian diet predicted a decrease in HDL-C in both GG and GA + AA individuals belonging to the exercise (*β* = −7.9361 and *β* = −4.5577, respectively, *p* < 0.05) and no exercise (*β* = −5.1297 and β = −4.6986, *p* < 0.0001) groups, respectively (Table 3). With non-vegetarian diet plus no exercise as the reference group, vegetarian diet/no exercise was associated with a 5.1514 mg/dl reduction in HDL-C among GG carriers (*β* = −5.1514, *p* < 0.0001) and a 4.8426 mg/dl decrease (*β* = −4.8426, *p* < 0.0001) among GA + AA carriers (Table 4). Likewise, a vegetarian diet/regular exercise was associated with a 6.5552 mg/dl reduction in HDL-C among GG carriers (*β* = −6.5552, *p* < 0.0001), and a 2.8668 mg/dl decrease in HDL-C among those with GA + AA genotypes. Regular exercise/non-vegetarian diet predicted a 1.3135 mg/dl (*β* = 1.3135, *p* < 0.0001) and 1.4301 mg/dl (*β* = 1.4301 *p* < 0.0002) reduction in HDL-C among GG and GA + AA individuals, respectively.

## 4. Discussion

As far as we understand, this study is the first to determine the relationship between HDL-C and a vegetarian diet together with exercise exposure in Taiwanese adults, based on *MTHFR* rs1801133 polymorphism. We found that exposure to exercise was linked to higher HDL-C levels in GG and GG + AA individuals, whereas the intake of vegetarian diets was associated with lower levels. Compared to a vegetarian diet alone, vegetarian diets combined with regular exercise led to much lower HDL-C levels among GG individuals, and comparatively higher levels among those with GA + AA genotype. 

We believe that several studies have already examined associations between HDL-C, exercise and diets. However, such associations have not been analyzed based on the *MTHFR* rs1801133 polymorphism as we have done in the current study. In a systemic review [18], the rs1801133 polymorphic variant showed a strong association with lower HDL-C. The standard mean difference (SMD) was −0.10. In our study, we observed similar results in both GG and GA + AA individuals who were on vegetarian diets. As might have been expected, exercise exposure showed positive associations with HDL-C in individuals carrying both genotypes. The mechanism behind these associations needs to be further investigated. Despite this, exercise has been demonstrated to increase the expression of liver ATP-binding cassette transporters A-1, that help to strengthen the reverse cholesterol transport system as mentioned above. Exercise in combination with diet has been associated with biomarkers of metabolic disorders and variations in body composition [28]. However, the effective outcome of the combination was based on the exercise type.

In the current study, men had lower HDL-C compared to women, no matter the genotype. We also found that overweight/obese individuals had lower HDL-C levels than normal-weight individuals with both genotypes. Besides, high levels of triglycerides (TG) also predicted lower HDL-C. These findings are in line with those previously reported by Zhi and his team, wherein overweight and obesity were strongly associated with a higher TG but lower HDL-C in individuals with *MTHFR* rs1801133 genotypes [24]. Rs1801133 polymorphism has also been suggested to increase obesity risk in both adolescents and children [29]. Serum uric acid was also included in our model. We found that the uric acid levels did not differ greatly among the samples (Table S1). Our analyses also indicated that higher (i.e., men, ≥7 mg/dl and women, ≥6 mg/dl) compared to lower uric acid (men, <7 mg/dl and women, <6 mg/dl) was associated with lower HDL-C, no matter the genotype. Negative associations between serum uric acid and HDL-C have been previously described [30,31]. Of note, polymorphic variants were not included in the model as we have done.

It appears reasonable to state that exercise exposure is one of the great ways for vegetarians to boost their HDL cholesterol [16]. However, our investigation into this area suggests that the association of HDL-C with a vegetarian diet in combination with exercise appears to differ for each Taiwanese individual based on the genotype. Despite these findings, several shortfalls need to be considered. First, recall bias is possible in our study, considering that data on exercise patterns were collected based on self-report. Second, we did not have data on the average daily intake of macro and micronutrients. In addition, we could not present data that indicated how long participants might have followed a vegetarian diet. Moreover, our analyses had a total of 443 vegetarians, 35 of whom were vegans. Because of this, we could not perform a subgroup analysis separately for the vegans. However, we hope that our findings will serve as a base for future studies on HDL-C and the associated lifestyle and dietary changes.

## 5. Conclusions

In conclusion, our findings revealed that exercise exposure predicted a higher HDL-C among *MTHFR* rs1801133 GG and GA + AA individuals. A vegetarian diet alone was associated with lower HDL-C. However, the inclusion of regular exercise resulted in much lower levels among GG individuals, whereas levels in GA + AA individuals were somewhat higher. In summary, a vegetarian diet in combination with exercise predicted an increase in HDL-C levels only among individuals with *MTHFR* rs1801133-GA + AA genotype in Taiwan. These findings point to the likelihood that combination lifestyle therapies might not serve as an alternative means of boosting cholesterol levels in Taiwanese adults with the rs1801133-GG genotype.

## Figures and Tables

**Table 1:** Demographic characteristics of participants grouped by rs1801133 genotypes.

Variable	rs1801133-GG (*n* = 5016)		rs1801133-GA + AA (*n* = 4239)		*p*-Value
	N	Mean HDL-C (SE)	N	Mean HDL-C (SE)	
Diet type					0.9828
Non-vegetarian	4542	54.056 (0.198)	3842	54.010 (0.214)	
Former vegetarian	232	54.297 (0.953)	196	53.510 (0.960)	
Vegetarian	242	49.591 (0.707)	201	50.010 (0.867)	
Exercise					0.8657
No	2909	53.146 (0.244)	2451	52.993 (0.262)	
Yes	2107	54.826 (0.294)	1788	54.900 (0.320)	
Sex					0.5662
Women	2690	58.455 (0.255)	2248	58.363 (0.280)	
Men	2326	48.529 (0.234)	1991	48.642 (0.249)	
Age (years)					0.9905
30–40	1313	53.998 (0.364)	1102	53.786 (0.394)	
41–50	1407	53.684 (0.352)	1182	53.483 (0.387)	
51–60	1437	53.446 (0.353)	1224	54.227 (0.377)	
61–70	859	54.582 (0.462)	731	53.603 (0.498)	
TG (mg/dl)					0.0046
<150	3896	56.711 (0.207)	3395	56.300 (0.223)	
≥150	1120	43.907 (0.273)	844	43.732 (0.297)	
LDL-C (mg/dl)					0.6191
<130	3069	54.185 (0.257)	2615	54.001 (0.270)	
≥130	1947	53.327 (0.265)	1624	53.470 (0.304)	
WHR					0.0770
Men < 0.9; women < 0.85 (ref)	2757	56.636 (0.259)	2252	56.206 (0.288)	
Men ≥ 0.9; women ≥ 0.85	2259	50.455 (0.255)	1987	51.067 (0.273)	
Body mass index, kg/m^2^					0.0233
18.5–23.9 (ref)	2489	58.039 (0.265)	1969	57.837 (0.298)	
<18.5	128	66.828 (1.278)	108	65.204 (1.391)	
24–26.9	1443	50.338 (0.309)	1298	51.276 (0.330)	
≥27	956	46.517 (0.319)	864	46.953 (0.360)	
Body fat (%)					0.1882
Men < 25; women < 30 (ref)	2711	55.969 (0.268)	2233	55.456 (0.288)	
Men ≥ 25; women ≥ 30	2305	51.363 (0.251)	2006	51.951 (0.280)	
Smoking					0.3176
Nonsmokers	3893	55.378 (0.213)	3281	55.468 (0.233)	
Former smokers	568	49.889 (0.513)	516	48.953 (0.476)	
Current smokers	555	47.205 (0.500)	442	47.048 (0.549)	
Alcohol intake					0.2371
nondrinkers	4495	54.211 (0.197)	3820	54.199 (0.216)	
Former drinkers	160	45.531 (0.821)	110	48.036 (1.005)	
Current drinkers	361	53.066 (0.755)	309	50.883 (0.658)	
Coffee drinking					0.4381
No	3333	53.361 (0.227)	2849	53.448 (0.247)	
Yes	1683	54.825 (0.332)	1390	54.514 (0.356)	
Uric acid, mg/dl					0.0334
men < 7, women < 6 (ref)	3851	55.528 (0.217)	3174	55.665 (0.236)	
men ≥ 7, women ≥ 6	1165	48.311 (0.328)	1065	48.230 (0.350)	

SE: standard error; WHR: waist-to-hip ratio; BMI: Body mass index; TG: triglyceride; LDL-C: Low-density lipoprotein cholesterol; HDL-C: High-density lipoprotein cholesterol.

**Table 2:** Regression coefficients of lifestyle variables associated with HDL-C according to rs1801133 genotypes.

Variables	rs1801133-GG		rs1801133-GA + AA	
	*β*	*p*-Value	*β*	*p*-Value
Diet type (ref: Non-vegetarian)				
Former vegetarian	−0.1917	0.7918	−0.3995	0.6222
Vegetarian	−6.2793	<0.0001	−4.6359	<0.0001
*p*-trend	<0.0001		<0.0001	
Exercise (ref: No)				
Yes	1.0508	0.0015	1.4011	0.0001
Sex (ref: women)				
Men	−7.9509	<0.0001	−7.2069	<0.0001
Age (ref: 30–40)				
41–50	0.5879	0.1630	0.5495	0.2453
51–60	1.0893	0.0131	1.2023	0.0149
61–70	1.7187	0.0009	1.0543	0.0673
TG (ref: <150)				
≥150	−8.2129	<0.0001	−8.1116	<0.0001
LDL-C (ref: <130)				
≥130	1.1095	0.0005	1.4316	0.0001
WHR (ref: men < 0.9; women < 0.85)				
Men ≥ 0.9; women ≥ 0.85	−2.6110	<0.0001	−2.4022	<0.0001
Body mass index, kg/m^2^ (ref: 18.5–23.9)				
<18.5	6.2828	<0.0001	5.7646	<0.0001
24–26.9	−3.6743	<0.0001	−3.1293	<0.0001
≥27	−4.5426	<0.0001	−5.0012	<0.0001
Body fat (ref: Men < 25; women < 30)				
Men ≥ 25; women ≥ 30	−1.9773	<0.0001	−0.6996	0.1201
Smoking (ref: Nonsmokers)				
Former smokers	−0.0117	0.9824	−0.9565	0.0988
Current smokers	−2.7451	<0.0001	−1.9437	0.0019
Alcohol intake (ref: Nondrinkers)				
Former drinkers	−1.0938	0.2265	−0.4513	0.6793
Current drinkers	6.0734	<0.0001	3.5075	<0.0001
Coffee drinking (ref: No)				
Yes	0.7415	0.0230	0.5075	0.1650
Uric acid, mg/dl (ref: men < 7, women <6)				
men ≥ 7, women ≥ 6	−1.5541	0.0001	−2.1708	<0.0001

**Table 3:** Association of vegetarian diet with HDL-C based on rs1801133 genotypes and exercise exposure.

Variables	rs1801133-GG				rs1801133-GA + AA			
	No Exercise		Exercise		No Exercise		Exercise	
	*β*	*p*-Value	*β*	*p*-Value	*β*	*p*-Value	*β*	*p*-Value
Diet type (ref: Non-vegetarian)								
Former vegetarian	0.8314	0.3476	−2.0019	0.1127	0.0519	0.9581	−1.3033	0.3485
Vegetarian	−5.1297	<0.0001	−7.9361	<0.0001	−4.6986	<0.0001	−4.5577	0.0008
*p*-trend	<0.0001		<0.0001		<0.0001		0.0007	
Sex (ref: women)								
Men	−8.1078	<0.0001	−7.7185	<0.0001	−6.6446	<0.0001	−8.0869	<0.0001
Age (ref: 30–40)								
41–50	0.4769	0.3248	0.7870	0.3552	1.0190	0.0585	−0.8985	0.3522
51–60	1.1302	0.0369	0.9529	0.2370	1.9337	0.0015	−0.1497	0.8691
61–70	1.5450	0.0371	1.6555	0.0523	0.3052	0.6980	0.6124	0.5294
TG (ref: <150)								
≥150	−7.7074	<0.0001	−8.9542	<0.0001	−7.7816	<0.0001	−8.6927	<0.0001
LDL-C (ref: <130)								
≥130	1.0732	0.0102	1.1117	0.0276	2.1314	<0.0001	0.4474	0.4294
WHR (ref: Men < 0.9; women < 0.85)								
Men ≥ 0.9; women ≥ 0.85	−2.6070	<0.0001	−2.6692	<0.0001	−2.5188	<0.0001	−2.4311	0.0001
Body mass index, kg/m^2^ (ref: 18.5–23.9)								
BMI < 18.5	6.1337	<0.0001	6.6755	0.0002	4.6449	0.0002	9.2076	<0.0001
24–26.9	−3.6149	<0.0001	−3.7077	<0.0001	−3.2793	<0.0001	−2.9512	<0.0001
≥27	−4.0139	<0.0001	−5.3350	<0.0001	−5.3062	<0.0001	−4.5617	<0.0001
Body fat (ref: Men < 25; women < 30)								
Men ≥ 25; women ≥ 30	−2.2468	<0.0001	−1.6160	0.0119	−0.7847	0.1727	−0.6886	0.3386
Smoking (ref: Nonsmokers)								
Former smokers	−0.1570	0.8269	0.1372	0.8636	−1.0469	0.1701	−0.5860	0.5120
Current smokers	−2.8800	<0.0001	−2.5990	0.0096	−2.6090	0.0004	−1.0954	0.3494
Alcohol intake (ref: Nondrinkers)								
Former drinkers	−0.7260	0.5865	−1.4799	0.2390	−1.3610	0.3676	0.6249	0.6957
Current drinkers	6.5085	<0.0001	5.3821	<0.0001	4.8254	<0.0001	1.8587	0.0967
Coffee drinking (ref: No)								
Yes	0.9987	0.0167	0.3849	0.4616	0.5014	0.2790	0.5678	0.3391
Uric acid, mg/dl (ref: men < 7, women < 6)								
men ≥ 7, women ≥ 6	−1.3670	0.0065	−1.7314	0.0044	−1.8402	0.0006	−2.7109	0.0001

β: beta coefficient; WHR: waist-to-hip ratio; BMI: Body mass index: TG triglyceride; LDL-C: Low-density lipoprotein cholesterol; HDL-C: High-density lipoprotein cholesterol.

**Table 4:** Exercise exposure together with vegetarian diets and their association with HDL-C.

Variables	rs1801133-GG		rs1801133-GA + AA	
	*β*	*p*-Value	*β*	*p*-Value
Exercise and Diet type (ref: no exercise/non-vegetarian)				
No exercise/Former vegetarian	0.8068	0.3724	0.0541	0.9577
No exercise/Vegetarian	−5.1514	<0.0001	−4.8426	<0.0001
Regular exercise/non-vegetarian	1.3135	0.0001	1.4301	0.0002
Regular exercise/Former vegetarian	−0.6585	0.5862	0.2568	0.8464
Regular exercise/Vegetarian	−6.5552	<0.0001	−2.8668	0.0273
Sex (ref: women)				
Men	−7.9224	<0.0001	−7.2075	<0.0001
Age (ref: 30–40)				
41–50	0.6059	0.1504	0.5663	0.2317
51–60	1.0645	0.0153	1.2121	0.0142
61–70	1.6897	0.0011	1.0612	0.0656
TG (ref: <150)				
≥150	−8.2103	<0.0001	−8.1141	<0.0001
LDL-C (ref: <130)				
≥130	1.1034	0.0006	1.4310	0.0001
WHR (ref: Men < 0.9; women < 0.85)				
Men ≥ 0.9; women ≥ 0.85	−2.6201	<0.0001	−2.4058	<0.0001
Body mass index, kg/m^2^ (ref: 18.5–23.9)				
<18.5	6.3076	<0.0001	5.7872	<0.0001
24–26.9	−3.6710	<0.0001	−3.1217	<0.0001
≥27	−4.5409	<0.0001	−4.9955	<0.0001
Body fat (ref: Men < 25; women < 30)				
Men ≥ 25; women ≥ 30	−1.9766	<0.0001	−0.6998	0.1200
Smoking (ref: Nonsmokers)				
Former smokers	−0.0321	0.9518	−0.9705	0.0942
Current smokers	−2.7519	<0.0001	−1.9387	0.0020
Alcohol intake (ref: Nondrinkers)				
Former drinkers	−1.1352	0.2093	−0.4382	0.6882
Current drinkers	6.0677	<0.0001	3.5075	<0.0001
Coffee drinking (ref: No)				
Yes	0.7495	0.0215	0.5110	0.1625
Uric acid, mg/dl (ref: men < 7, women < 6)				
men ≥ 7, women ≥ 6	−1.5389	0.0001	−2.1757	<0.0001

β: beta coefficient; WHR: waist-to-hip ratio; BMI: Body mass index: TG triglyceride; LDL-C: Low-density lipoprotein cholesterol; HDL-C: High-density lipoprotein cholesterol.

## Supplementary Materials

The following are available online at https://www.mdpi.com/2072-6643/12/6/1564/s1, Table S1: Mean serum uric acid (mg/dl) levels of the sample.

## References

[1] Mann S., Beedie C., Jimenez A. (2014). Differential effects of aerobic exercise, resistance training and combined exercise modalities on cholesterol and the lipid profile: Review, synthesis and recommendations. Sports Med..

[2] Yusuf S., Hawken S., Ounpuu S., Dans T., Avezum A., Lanas F., McQueen M., Budaj A., Pais P., Varigos J. (2004). Effect of potentially modifiable risk factors associated with myocardial infarction in 52 countries (the INTERHEART study): Case-control study. Lancet.

[3] März W., Kleber M.E., Scharnagl H., Speer T., Zewinger S., Ritsch A., Parhofer K.G., von Eckardstein A., Landmesser U., Laufs U. (2017). HDL cholesterol: Reappraisal of its clinical relevance. Clin. Res. Cardiol..

[4] Parhofer K.G. (2015). Interaction between glucose and lipid metabolism: More than diabetic dyslipidemia. Diabetes Metab. J..

[5] Shor R., Wainstein J., Oz D., Boaz M., Matas Z., Fux A., Halabe A. (2008). Low HDL levels and the risk of death, sepsis and malignancy. Clin. Res. Cardiol..

[6] Yeh M.-C., Glick-Bauer M., Watson R.R., Preedy V.R. (2016). Chapter 8—Vegetarian diets and disease outcomes. Fruits, Vegetables, and Herbs.

[7] Picasso M.C., Lo-Tayraco J.A., Ramos-Villanueva J.M., Pasupuleti V., Hernandez A.V. (2019). Effect of vegetarian diets on the presentation of metabolic syndrome or its components: A systematic review and meta-analysis. Clin. Nutr..

[8] Huang Y.-W., Jian Z.-H., Chang H.-C., Nfor O.N., Ko P.-C., Lung C.-C., Lin L.-Y., Ho C.-C., Chiang Y.-C., Liaw Y.-P. (2014). Vegan diet and blood lipid profiles: A cross-sectional study of pre and postmenopausal women. BMC Women’s Health.

[9] Wang F., Zheng J., Yang B., Jiang J., Fu Y., Li D. (2015). Effects of vegetarian diets on blood lipids: A systematic review and meta-analysis of randomized controlled trials. J. Am. Heart Assoc..

[10] Yokoyama Y., Levin S.M., Barnard N.D. (2017). Association between plant-based diets and plasma lipids: A systematic review and meta-analysis. Nutr. Rev..

[11] Sambol S.Z., Glišić M.O., Bašić N.M., Dvornik Š, Grahovac B., Mihić S.S., Štimac D. (2017). Influence of Dietary Pattern and Methylentetrahydrofolate Reductase C677t Polymorphism on the Plasma Homocysteine Level Among Healthy Vegetarians and Omnivores. Hrana Zdr. Boles. Znan. Stručni Časopis Za Nutr. Dijetetiku.

[12] Kelley G.A., Kelley K.S. (2012). Effects of diet, aerobic exercise, or both on non-HDL-C in adults: A meta-analysis of randomized controlled trials. Cholesterol.

[13] Drygas W., Kostka T., Jegier A., Kuski H. (2000). Long-term effects of different physical activity levels on coronary heart disease risk factors in middle-aged men. Int. J. Sports Med..

[14] Larrydurstine J., Haskell W.L. (1994). Effects of exercise training on plasma lipids and lipoproteins. Exerc. Sport Sci. Rev..

[15] MayoClinic.Com HDL Cholesterol: How to Boost Your ‘Good’ Cholesterol. https://www.mayoclinic.org/diseases-conditions/high-blood-cholesterol/in-depth/hdl-cholesterol/art-20046388?pg=2.

[16] Wang Y., Xu D. (2017). Effects of aerobic exercise on lipids and lipoproteins. Lipids Health Dis..

[17] Williams P.T. (1997). Interactive effects of exercise, alcohol, and vegetarian diet on coronary artery disease risk factors in 9242 runners: The National Runners’ Health Study. Am. J. Clin. Nutr..

[18] Luo Z., Lu Z., Muhammad I., Chen Y., Chen Q., Zhang J., Song Y. (2018). Associations of the MTHFR rs1801133 polymorphism with coronary artery disease and lipid levels: A systematic review and updated meta-analysis. Lipids Health Dis..

[19] Christensen K.E., Mikael L.G., Leung K.-Y., Lévesque N., Deng L., Wu Q., Malysheva O.V., Best A., Caudill M.A., Greene N.D. (2015). High folic acid consumption leads to pseudo-MTHFR deficiency, altered lipid metabolism, and liver injury in mice. Am. J. Clin. Nutr..

[20] Kukrele P., Sharma R. (2016). High homocysteine level-An important risk factor for coronary artery disease in vegetarians. J. Assoc. Physicians India.

[21] Frosst P., Blom H., Milos R., Goyette P., Sheppard C.A., Matthews R., Boers G., Den Heijer M., Kluijtmans L., Van Den Heuve L. (1995). A candidate genetic risk factor for vascular disease: A common mutation in methylenetetrahydrofolate reductase. Nat. Genet..

[22] Nie Y., Gu H., Gong J., Wang J., Gong D., Cong X., Chen X., Hu S. (2011). Methylenetetrahydrofolate reductase C677T polymorphism and congenital heart disease: A meta-analysis. Clin. Chem. Lab. Med. (Cclm).

[23] Tanaka T., Scheet P., Giusti B., Bandinelli S., Piras M.G., Usala G., Lai S., Mulas A., Corsi A.M., Vestrini A. (2009). Genome-wide association study of vitamin B6, vitamin B12, folate, and homocysteine blood concentrations. Am. J. Hum. Genet..

[24] Zhi X., Yang B., Fan S., Wang Y., Wei J., Zheng Q., Sun G. (2016). Gender-specific interactions of MTHFR C677T and MTRR A66G polymorphisms with overweight/obesity on serum lipid levels in a Chinese Han population. Lipids Health Dis..

[25] Iemitsu M., Murakami H., Sanada K., Yamamoto K., Kawano H., Gando Y., Miyachi M. (2010). Lack of carotid stiffening associated with MTHFR 677TT genotype in cardiorespiratory fit adults. Physiol. Genom..

[26] Chang S.-L., Lee K.-J., Nfor O.N., Chen P.-H., Lu W.-Y., Ho C.C., Lung C.-C., Chou M.-C., Liaw Y.-P. (2020). Vegetarian Diets along with Regular Exercise: Impact on High-Density Lipoprotein Cholesterol Levels among Taiwanese Adults. Medicina.

[27] Tantoh D.M., Lee K.-J., Nfor O.N., Liaw Y.-C., Lin C., Chu H.-W., Chen P.-H., Hsu S.-Y., Liu W.-H., Ho C.-C. (2019). Methylation at cg05575921 of a smoking-related gene (AHRR) in non-smoking Taiwanese adults residing in areas with different PM 2.5 concentrations. Clin. Epigenetics.

[28] Clark J.E. (2015). Diet, exercise or diet with exercise: Comparing the effectiveness of treatment options for weight-loss and changes in fitness for adults (18–65 years old) who are overfat, or obese; systematic review and meta-analysis. J. Diabetes Metab. Disord..

[29] Borai I.H., Soliman A.F., Ahmed H.M., Ahmed G.F., Kassim S.K. (2018). Association of MTHFR C677T and ABCA1 G656A polymorphisms with obesity among Egyptian children. Gene Rep..

[30] Son M., Seo J., Yang S. (2020). Association between dyslipidemia and serum uric acid levels in Korean adults: Korea National Health and Nutrition Examination Survey 2016–2017. PLoS ONE.

[31] Ali N., Rahman S., Islam S., Haque T., Molla N.H., Sumon A.H., Kathak R.R., Asaduzzaman M., Islam F., Mohanto N.C. (2019). The relationship between serum uric acid and lipid profile in Bangladeshi adults. BMC Cardiovasc. Disord..

