# Supplementary material for: Combination of Exercise and Vegetarian Diet: Relationship with High Density-Lipoprotein Cholesterol in Taiwanese Adults Based on MTHFR rs1801133 Polymorphism
Source: Nutrients. 2020 May 27;12(6):1564. doi: 10.3390/nu12061564 (PMC7352486; doi:10.3390/nu12061564)
Supplement: Supplementary file 1 [file nutrients-12-01564-s001.pdf]

Supplementary Table 1. Mean serum uric acid (mg/dl) levels of the sample

	rs1801133-GG	rs1801133-GA+AA	*p*-value
	Mean uric acid (SE)	Mean uric acid (SE)	
Diet type			
Non vegetarian	5.622 (0.022)	5.641 (0.024)	0.5622
Former vegetarian	5.428 (0.097)	5.581 (0.113)	0.3038
Vegetarian	5.057 (0.086)	5.280 (0.103)	0.0936

SE: standard error.
